# Transmission dynamics: Data sharing in the COVID‐19 era

**DOI:** 10.1002/lrh2.10235

**Published:** 2020-06-28

**Authors:** Randi E. Foraker, Albert M. Lai, Thomas G. Kannampallil, Keith F. Woeltje, Anne M. Trolard, Philip R. O. Payne

**Affiliations:** ^1^ Institute for Informatics Washington University School of Medicine St. Louis Missouri USA; ^2^ Department of Medicine Washington University School of Medicine St. Louis Missouri USA; ^3^ Department of Anesthesiology Washington University School of Medicine St. Louis Missouri USA; ^4^ BJC HealthCare St. Louis Missouri USA

**Keywords:** collaboration, data sharing, healthcare delivery, population health

## Abstract

**Problem:**

The current coronavirus disease 2019 (COVID‐19) pandemic underscores the need for building and sustaining public health data infrastructure to support a rapid local, regional, national, and international response. Despite a historical context of public health crises, data sharing agreements and transactional standards do not uniformly exist between institutions which hamper a foundational infrastructure to meet data sharing and integration needs for the advancement of public health.

**Approach:**

There is a growing need to apply population health knowledge with technological solutions to data transfer, integration, and reasoning, to improve health in a broader learning health system ecosystem. To achieve this, data must be combined from healthcare provider organizations, public health departments, and other settings. Public health entities are in a unique position to consume these data, however, most do not yet have the infrastructure required to integrate data sources and apply computable knowledge to combat this pandemic.

**Outcomes:**

Herein, we describe lessons learned and a framework to address these needs, which focus on: (a) identifying and filling technology “gaps”; (b) pursuing collaborative design of data sharing requirements and transmission mechanisms; (c) facilitating cross‐domain discussions involving legal and research compliance; and (d) establishing or participating in multi‐institutional convening or coordinating activities.

**Next steps:**

While by no means a comprehensive evaluation of such issues, we envision that many of our experiences are universal. We hope those elucidated can serve as the catalyst for a robust community‐wide dialogue on what steps can and should be taken to ensure that our regional and national health care systems can truly learn, in a rapid manner, so as to respond to this and future emergent public health crises.

## 1 PROBLEM

Access to large‐scale and multimodal data is essential to creating an environment where data‐driven approaches to research, health care delivery, and population health are the norm, rather than an exception.^1^ In many cases, data can be obtained and corresponding analyses conducted using local resources, simplifying the technical and regulatory dimensions of such efforts. However, solutions to the COVID‐19 pandemic require data extending beyond a single health system or institution. There is an urgent imperative to obtain and integrate diverse and multi‐institutional data for timely, data‐driven insights that reflect the scope of the pandemic.^2^

As a result of lessons learned from tracking Ebola, Zika, and other outbreaks, the Centers for Disease Control and Prevention (CDC) outlined a strategic plan for surveillance.^3^ The plan focused on (a) establishing data standards, (b) decreasing unnecessary and redundant reporting burden, and (c) reducing the number of stand‐alone systems. To achieve these goals and facilitate data sharing, it is imperative to work across and between traditional organizational boundaries. Such work invariably involves navigating a myriad of regulatory and infrastructure issues that are substantially influenced by local, regional, and national political forces.^4^, ^5^, ^6^ Ultimately, in situations such as the current pandemic, the health of our cities, states, and our nation becomes a shared responsibility, wherein the exchange and integration of data is a foundational resource need.

The CDC framework has not been realized in the “real world” and thus we need to redouble efforts to learn from the current epidemic and ensure we build durable data sharing infrastructure to help respond to ongoing and future public health threats. Herein we describe a set of perspectives concerning the practical issues encountered when seeking to address such shared responsibilities, and the ensuing exchange and integration of data at all relevant levels, from local to national and international‐scale public health and research initiatives. In all such cases, we have found that in order to achieve our objectives, it has been necessary and desirable to:

1. *Identify and fill technology “gaps”* relevant to data sharing efforts, using existing capabilities, and to reduce the time to implementation of mission‐critical data sharing efforts;
2. Engage, to the extent possible, in the *collaborative design of data sharing requirements and transmission mechanisms*, to reduce redundancies and establish economies‐of‐scale;
3. *Facilitate cross‐domain discussions involving legal and research compliance* professionals to identify pathways for new or novel data sharing efforts to be appropriately and effectively managed from a regulatory perspective; and
4. *Establish or participate in multi‐institutional convening or coordinating activities*, to create and sustain data‐sharing communities of practice comprised of organizations with complementary needs, expertise, and capabilities.

Our experience has been a mixture of both successes and challenges. Our challenges, namely in creating programs or initiatives that have proven difficult, are a function of: (1) inadequate technology and data infrastructure; (2) duplication of data requests and inconsistent technical requirements that impose problematic technical “costs” associated with engaging in such sharing; and (3) insufficient coordination of efforts by responsible entities or authorities. These challenges represent an important body of “lessons learned” that should be addressed both in the near‐term, and, in some instances, in longer‐term contexts, such that we will be better prepared to operate a dynamic and data‐driven response to future pandemics or other public health threats. Proposed approaches follow.

## 2 SHORT‐TERM APPROACHES

Addressing the challenges presented by the COVID‐19 pandemic requires comprehensive data assets, spanning institutions and geographies and thus provide “multi‐scale” lessons learned.^2^, ^7^ Key to harnessing the data needed to answer these critical questions is an understanding of the “transmission dynamics” associated with these data elements as we work with our regional, state‐wide, and national partners. Our work at Washington University and BJC HealthCare in response to the COVID‐19 pandemic has leveraged a variety of data types and sources, including electronic health records (EHR), laboratory information systems, population health indicators, and numerous operational and facilities related data resources, all available at different levels of scales and granularities. The following is a description of our short‐term solutions to the challenges that presented themselves in the early weeks of the pandemic.

### 2.1 Challenge: Inadequate technology and data infrastructure

Much of our early, regional‐scale data sharing and analytics activity involved working with local public health departments to expand and enhance high‐level required reporting practices and disease surveillance capabilities in response to COVID‐19. The St. Louis metropolitan statistical area spans two states and includes 15 counties, and as a result of this geopolitical fragmentation, although local health departments have a shared database maintained by the state to use for notifiable conditions, they do not have a shared data infrastructure that is capable of handling the pace of pandemic data flow or a common baseline suite of data management tools for use in support of activities such as case management, contact tracing, or epidemiological modeling. This fragmentation has been particularly evident when seeking to execute the orders and mandates for the reporting of health system data in response to the public health threat posed by COVID‐19 that have been issued by both state and local government agencies and officials in accordance with relevant statutes.^8^

**FIGURE 1:**
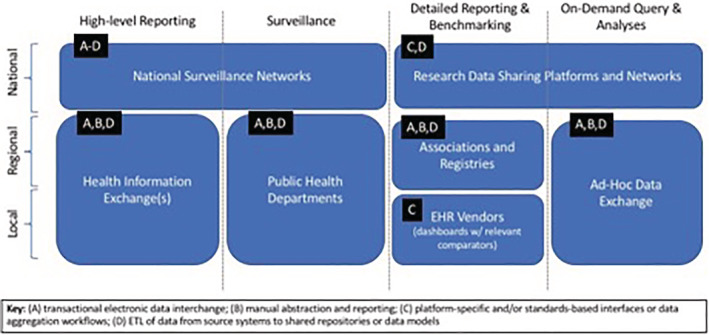
COVID data sharing efforts at Washington University and BJC HealthCare, illustrating a diverse set of scales, use cases, and transactional standards

To meet immediate needs, we have established a COVID‐19 data “commons” comprising regional data for operational and research use. These data have been shared in a number of capacities at the local, regional, national, and international levels, for the purposes of high‐level reporting, disease surveillance, detailed reporting and benchmarking, and on‐demand query, and analysis (Figure 1). The “end‐points” for such data sharing activities include a number of potentially useful functions and systems, including, but not limited to: institutional dashboards and geospatial visualizations, regional associations and registries, national surveillance and data sharing networks, and ad hoc data exchanges at local, regional, national, and international levels in order to inform “just‐in‐time” operational and research‐oriented decision‐making.^4^, ^7^, ^9^, ^10^

### 2.2 Challenge: Duplication of data requests and inconsistent technical requirements

Data requests vary by health departments, time scales, and data elements that are involved. Transmissions may occur via fax, a secure file transfer protocol, or a health level seven (HL7) interface. This lack of consistency across requests has complicated data sharing efforts as multiple data queries and exports must be conducted for realizing each request. Further, through our engagements with various local public health departments, we have also learned that their capacity for ingesting and analyzing these data varies widely, presenting substantial burdens to the timely use of such datasets and their effective application for reporting and analytical purposes.

After determining what data are potentially available, and for what purpose, it may be helpful to determine a minimum data set that is necessary to not overly burden health care provider organizations with data requests. During this crisis, such organizations are being barraged with requests for information, often through new regulatory mandates. Ideally, data should be the same for reporting to the city, county, and state public health officials to simplify the reporting burden. If the important work of conducting the data assessment is already completed, data recipients can better plan for the management and harmonization of the data elements that are transmitted. It is important to note that while a minimum data set may meet the immediate and ongoing need of public health surveillance, it will likely not be sufficient for predictive analytics or discovery.

Data sharing efforts span a variety of transactional‐ and data‐level standards and approaches, which collectively impose substantial costs and effort for participating in such data sharing regimes, including those at the international level, despite their considerable benefit from an analytical and decision‐making perspective. Mostly, these issues result from the aforementioned lack of shared data infrastructure, which resulted in a need to select de novo methods for each entity, rather than the use of extant tools/technologies to address specific data‐level requirements. In addition, there exists an absence of convening or coordinating bodies at the regional‐level working in the public or population health domains that are empowered to help harmonize or manage such data sharing requests across multiple entities.

To overcome these barriers, we engaged in a process of co‐design with the various public health departments to identify shared, common data elements and actionable data transfer formats or transactional standards, so as to simplify and systematize these requests. By performing this work at the intersection of the relevant healthcare provider organizations and on behalf of the public health departments, we have leveraged the unique expertise of our academic and operational biomedical and health informatics teams, and have been able to substantially simplify and expedite such data sharing, improving the speed, agility, and comprehensiveness of our regional public health infrastructure.

### 2.3 Challenge: Insufficient coordination of efforts by responsible entities or authorities

Meanwhile, we have seen a substantial demand for sharing of operational and clinical data between local healthcare provider organizations. For the most part, these data sharing requests are being made in support of detailed reporting and benchmarking efforts to facilitate demand management and capacity planning for potential COVID‐19‐related patient “surges”. However, despite the potential benefits of this type of data sharing and its ability to facilitate the production of common models of disease activity that enable providers to have increased “situational awareness,” the legal frameworks that govern such data sharing are both complex and are not necessarily well‐suited to a fast‐moving public health crisis.

Specifically, before shared analytic efforts can be undertaken by provider organizations and using a common data set that spans those entities, either: (a) a public health order must be issued, directing provider organizations to share their data with either health departments and/or an “honest broker”—either of whom can (potentially) reshare ensuing aggregate data with data contributors; and/or (b) a business associate agreement (BAA) can be established in a point‐to‐point manner to facilitate data sharing across and between providers or through an intermediate entity. In our case, we have positioned Washington University as a trusted intermediate entity or an “honest broker,” establishing a regional data “commons” for COVID‐19 analytics, and have pursued a dual‐track solution to enabling the population of that “commons” by: (a) working with our local public health departments to establish relevant public health orders that would direct participation in such a data sharing regime; as well as (b) executing BAAs across a consortium of collaborating health care provider organizations.

These parallel tracks to data sharing each require that data provider(s) and recipient(s) enumerate all types of data to be shared, the purpose of the data sharing effort (ie, operational or research‐focused), the time frame for its use, and when and if it can be disclosed for broader community benefit. All of the preceding areas represent potential points of negotiation for each pair‐wise agreement, thus introducing considerable complexity to the timely and resource‐efficient execution of the data sharing effort. We sought to expedite such BAAs and reduce the burden on our collaborators of participation in the regional data “commons” by defining a “minimum data set” for use by the partnering organizations that specified fewer than two dozen limited dataset elements that we had determined as central to answering our region's questions regarding COVID‐19 testing, symptoms, patient demographics and outcomes, and the overall geography of the pandemic.^2^, ^7^

Further, by creating and sharing templated documents for use among the various data partners, working in close consultation with legal experts, we were further able to expedite the execution of these agreements. Finally, it is important to note that with the engagement of a trusted intermediate entity to serve as the honest broker of these data (eg, Washington University), we were further able to expedite the implementation of the regional “commons” by reducing the number of agreements required. Specifically, instead of needing point‐to‐point BAAs for each pair‐wise combination of data sharing partners, we instead only needed one agreement per partner that was made with the honest broker.^11^

In addition to our work in the St. Louis region, we have investigated the expansion of the above‐mentioned concepts to a state‐wide framework, collecting data across the entire State of Missouri. To that end, in conjunction with the Governor's office of the State of Missouri, we entered into discussions with the four health information exchanges (HIEs) in Missouri to examine the feasibility of sourcing and aggregating data across the state, while minimizing burden on the individual provider organizations. We discovered that none of the HIEs retained substantial amounts of clinical data in their databases. However, the HIEs did regularly retain demographic information, a master patient index, and admission, discharge, and transfer information. This arrangement created a challenge with respect to aggregating data from across the state. One of the HIEs developed a mechanism such that for a given positive COVID‐19 test, they could query across their provider network for HL7 continuity of care documents and extract data elements of interest from those documents. Unfortunately, the other HIEs expressed not having the capability to perform similar tasks. Although the HIEs had agreements to enable data exchange between the HIEs, to date, no scalable solution has yet been found to aggregate all data statewide.

## 3 LONG‐TERM OUTCOMES: TOWARD A FRAMEWORK FOR EFFICIENT TRANSMISSION DYNAMICS

Informed by our experience, our team has conceptualized a pathway forward, whereby the engagement in various data sharing efforts relevant to our collective response to COVID‐19 can be made simpler, more efficient, and timely. Such an approach is ideally meant to support “speed‐to‐insights” as part of a very time‐sensitive pandemic response plan for all involved parties, while also reducing the technical and resource burdens associated with doing so, and hence, achieving *efficient transmission dynamics*.

**FIGURE 2:**
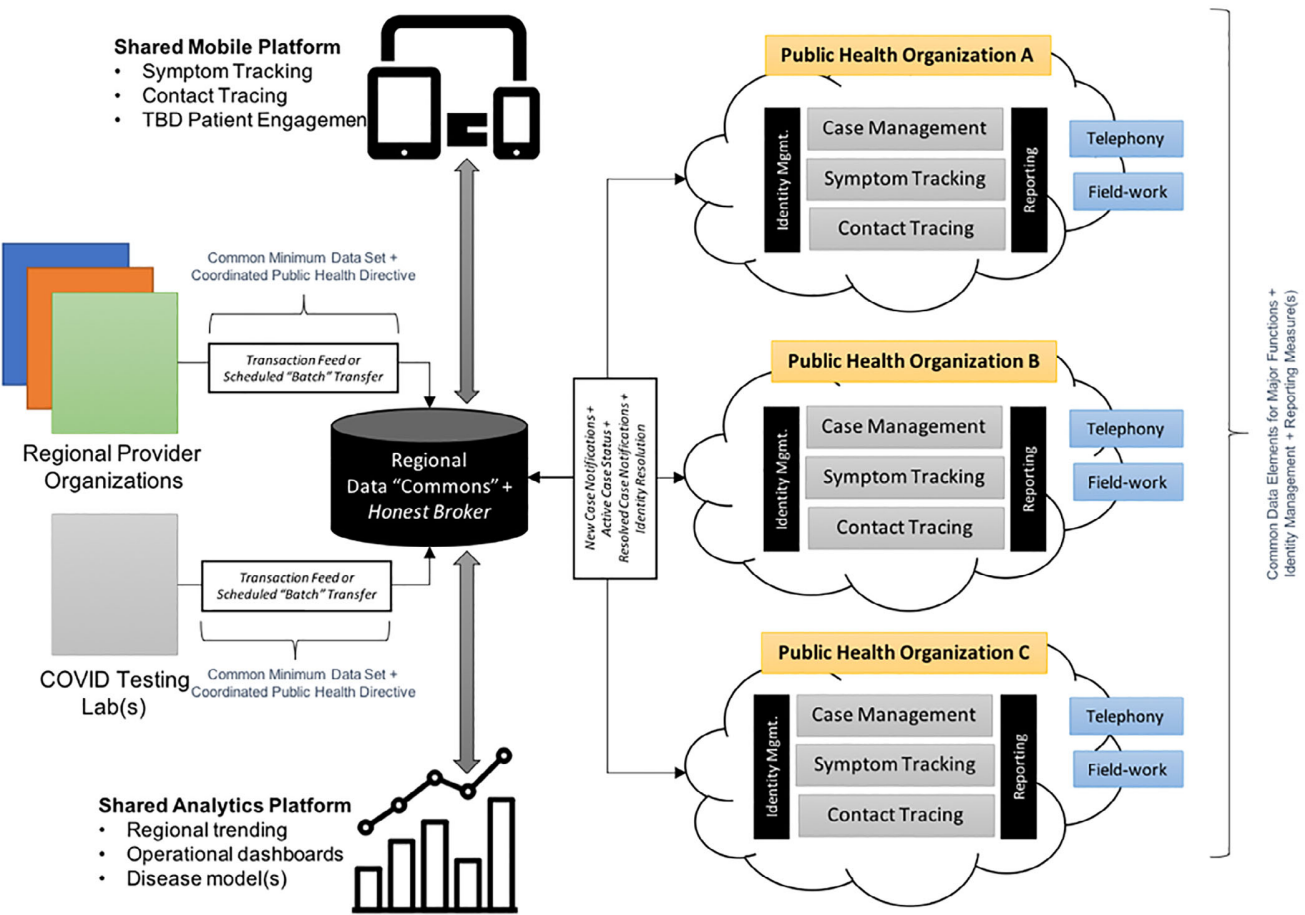
A conceptual model for a learning, digital public health data architecture which can be expanded in dimension to encapsulate individual institutions as well as collective efforts on the local, regional, national, and international levels

The basic “building block” for this approach is a functional unit that can be referred to as a learning digital public health data architecture, which is deployable across local, regional, national, and international domains (Figure 2). A learning health system provides a foundation for iterative data collection, integration, analysis, interpretation, and action with an eye toward continuous iteration.^12^, ^13^ This construct—applied to public health data—can enable and facilitate rapid‐cycle, collaborative data analytics involving multiple data partners and stakeholders. Note that while we present this construct as it relates to COVID‐19 data sharing, it is equally important to recognize that it can and should serve as the foundation for collaborative actions and goals which are central to the success of an ongoing, sustainable, population health strategy.^13^, ^14^ Attention to the scalability and sustainability of this approach to other public health threats from local to international scales is critical.

Extending the preceding “building blocks”, we can envision the creation of a national‐scale learning health system that can facilitate COVID‐19 focused data sharing and analytics, leveraging syntactic and semantic standards, as well as foundational common data models and data elements relevant to COVID‐19 phenotyping, such that a “network of networks” is created. As a result, local or regional efforts, from a data platform perspective, can contribute to the execution of national or international‐scale data efforts, without having to create disparate or otherwise additive data infrastructure, governance, and analysis tooling that may exist outside of the scope of what is needed to meet local needs (and thus represents an additional resource burden in order to participate effectively in such efforts).

Such an approach to creating a learning health care system is not new, and has been presented by Friedman, Lessard, and colleagues.^12^, ^14^ However, current experience has shown that we are still not using such frameworks when addressing real‐world problems. Our perspective is that such a missed opportunity is a function of the: (a) misalignment of incentives and funding mechanisms relative to achieving such a shared goal; (b) absence of suitable convening or coordinating bodies that can operate at a high‐enough level to harmonize and strategically align relevant data sharing and analytical efforts; and (c) insufficiency of current data infrastructure to scale and operate in the manner needed to enable this type of data sharing regime.

Such a regional digital public health data infrastructure can be linked with similar platforms across a state or nationally, to create scalable, reusable, sustainable infrastructure. In the sections below, we summarize specific recommendations based on high‐level lessons, as a means of advancing our current and future state relative to efficient transmission dynamics in a what has become an increasingly rapid learning health system that can succeed at multiple scales.

### 3.1 Identify and fill technology “gaps”

#### 3.1.1 Recommendation

Repurpose existing technologies and platforms to enable rapid data sharing.

As has been reported in the media, we found that there was a substantial gap in terms of public health informatics infrastructure, particularly surrounding data sharing and electronic data capture for case identification, contact tracing, and monitoring. We found ourselves with a choice to either develop “ideal” systems or to utilize already existing tooling to fill the gaps. Given the urgency of the need for this infrastructure, we focused initially on repurposing existing technologies and platforms to enable rapid data sharing and data collection, followed by quick iterations and continuous improvements on the use of these technologies. As we progress, and move toward the ideal state as shown in Figure 2, we would prefer to switch to existing or updated technologies that may be superior yet may take longer to implement.

At the beginning of our pandemic response, we sought to minimize new technology infrastructure and development activities, and started using real‐time reporting tools in our EHR. As time went on and the amount of data accumulated, we were able to move to using more sophisticated queries from our EHR database. As the analyses we need to conduct to support research and operations have evolved, we have begun to leverage our data warehousing infrastructure to support regional and national data sharing initiatives.

Another example of our need maximize reuse of extant tools and systems was that we encountered an urgent and substantial need to collect data not only to support operations at our academic health center, but also regionally to support the public health response. Although perhaps not necessarily designed for this purpose, we deployed our research data capture (REDCap) electronic data capture system in collaboration with local public health departments for their exposure monitoring, case identification, and contact tracing efforts. This system was initially deployed at the county level and is being adopted at other local public health departments in the region. As we move into what we expect to be a prolonged post‐peak era, with the luxury of time, we have begun to explore other more purpose‐built tools.

### 3.2 Pursue collaborative design of data sharing requirements and transmission mechanisms

#### 3.2.1 Recommendation

Determine the mechanisms and formats of data transmission.

Data sharing efforts can involve transactional electronic data exchange, manual abstraction and reporting, platform data aggregation, and extract, transform, and load functions that are often automated processes to populate shared repositories or data marts. Of note, as data transmission activities mature over the course of this pandemic, processes can evolve from manual abstraction to that of automated queries and transmission via secure file transfer protocols, HL7 feeds, or fast health care interoperability resources approaches. The mechanism of data transmission must adapt as standards‐based data workflows become more routine.

Given the time sensitivity of data in a public health crisis, flexibility in terms of data formats and mechanisms may be necessary. This is particularly salient given the large demands being placed upon individual health care provider organizations and local departments of health to request, send, and receive data. In addition, the mechanism used for data transfer may also be heavily dependent on the technical capabilities of the sending and receiving entities, both in terms of personnel and software infrastructure. In our discussions with HIEs, we uncovered that data transmissions were being conducted with a combination of mechanisms, such as secure emails, secure file transfer protocols, and HL7 interface feeds. With file‐based data transfer mechanisms, the data were also coming in a variety of formats, including comma separated values (csv), tab delimited, Microsoft Excel spreadsheets, and HL7 continuity of care documents. These data then need to be extracted and transformed into an actionable dataset.

#### 3.2.2 Recommendation

Harmonize, store, and retrieve data to enable analytic activities.

Analytic activities include, but are not limited to: reporting, surveillance, benchmarking, and on‐demand analyses. The unglamorous work of assessing and adjudicating data elements must be conducted before the data are entered into a data mart or other organized data management system. As these data are originating from fragmented systems, data cleaning and quality assurance activities will be invaluable to detect inconsistent or incomplete data before these data are relied upon for insights. Data definitions, even that of patients with a COVID‐19 positive test, can vary widely across data sources and discrepancies must be addressed early. Data feeds from collaborating entities must be transformed into analyzable datasets to meet the needs at local, regional, and national levels as shown in Figure 1.

One of the challenges early on was the lack of data standards [LOINC (logical observation identifiers names and codes)], CPT (current procedural or terminology), or international classification of diseases (ICD‐10) codes 10] as COVID‐19 emerged. Without these standards, there was substantial effort required to even extract the data of interest. As these standards did become available, substantial efforts remained in terms of harmonizing the data from various sources, both from combining data from before the existence of standards as well as making the value sets consistent. Simply determining whether or not a resulting COVID‐19 test was considered positive was a challenge. We saw values such as “detected,” “positive,” and “YES” in our data.

### 3.3 Facilitate cross‐domain discussions involving legal and research compliance

#### 3.3.1 Recommendation

Allow for iterative changes to data elements, agreements, transmission, and analysis.

Early in the pandemic, data elements were needed to determine the availability of COVID‐19 testing, the number of patients currently in the hospital and intensive care units, supply chain needs, and distribution of cases by geography. As our region reached its peak, our efforts partially shifted to simulate the post‐COVID‐19 era, determining the potential impacts of relaxing social distancing restrictions and predicting the next hotspot or cluster of new cases.

New data elements may be needed to supplement the prespecified minimum data set in order to explore these and other future analyses. We will continue to iterate on our regional learning health system in order to respond to the needs of our stakeholders. In order to enable this type of agility in compiling regional data assets, we must maintain regular contact with legal and compliance experts to navigate an ever‐changing landscape with respect to the required data elements.

### 3.4 Establish or participate in multi‐institutional convening or coordinating activities

#### 3.4.1 Recommendation: Create and sustain data‐sharing communities of practice comprised of organizations with complementary needs, expertise, and capabilities

In addition to regional and state‐wide data sharing endeavors, we have participated in several national‐level efforts. These have included basic and ad hoc exchanges of data in order to compare or validate predictive disease models via informal interaction with collaborators across the United States, to the development of COVID‐19‐specific disease registries and surveillance systems that enable both research and public health activities at a national level.^5^, ^9^ Broadly, such activities have leveraged some combination of: (a) existing reporting or electronic data interchange standards (such as those used by federal agencies, including the Department of Health and Human Services and the CDC); (b) vendor‐specific data sharing or interchange mechanisms, primarily situated within an EHR environment; and/or (c) research‐specific data federation platforms that had previously been established for a variety of clinical and translational research.

Most recently, we have launched efforts to create centralized data resources, held by either government entities and/or research collaboratives or professional associations, to augment preceding data transfer and sharing mechanisms, primarily for research and innovation purposes. Although such efforts address critical gaps in the extant infrastructure, they also represent a new source of technical burden for participation. Such burden takes many forms, including the effort required to extract, transform, and transmit data to such repositories, as well as the need to address complex data privacy and sharing policies and procedures that are needed to address both relevant legal frameworks as well as institutional concerns.^2^, ^9^

When viewed collectively, these national efforts remain nascent compared to the regional initiatives, which we believe is attributable to several factors, including: (a) overlapping policies and objectives of such efforts, creating a complex, and sometimes, competitive environment in which individual organizations have to identify priorities and/or partners based on local expectations and needs; (b) lack of harmonization between existing tools and technologies that could be used to enable such data sharing, including a strong disposition by many such programs to build new infrastructure de novo (at considerable cost in terms of both resources and timeliness) as opposed to leveraging existing infrastructure; and (c) complex, and often incompatible legal and regulatory frameworks and requirements that are not suited to enabling this type of collaborative work at‐scale.

Such barriers represent an existential threat to the creation of both public health capabilities and a clinical evidence‐base that will allow for an effective response to the COVID‐19 pandemic, and must be addressed in both the near‐ and longer‐term if we are going to protect the health of our citizens and balance public health and economic policy in a synergistic and mutually beneficial manner. It is our perspective that much higher levels of funding, coordination, and attention by policymakers, industry, health care provider organizations, and researchers alike will be needed to address such national‐scale issues. We are participating in the National COVID Cohort Collaborative, funded by the National Center for Advancing Translational Sciences at the National Institutes of Health. This collaborative includes several Center for Translational Science Award sites that are working through many of the same challenges outlined in this paper.

#### 3.4.2 Recommendation

Share best practices and resources across entities.

Not all participating entities will have the same level of data literacy nor data management expertise. We suggest those who have solved the puzzle of data retrieval, storage, and transmission should share best practices with respect to data queries, architecture, and sharing mechanics. These steps will serve to efficiently generate reports and summaries for different audiences including registries, governments, and the general public. It is also important to note that not all stakeholders are equipped with data analytics capacity. Thus, a centralized hub which is designated as the data recipient, manager, and analytics engine can help to accelerate the production of clear and actionable data reports and visualizations in this otherwise uncertain time.

## 4 CONCLUSIONS

As has been discussed throughout our report, the COVID‐19 pandemic has led to unprecedented need and demand for data sharing and collaborative analytics, working across and between traditional organizational boundaries. Such efforts have a variety of potential benefits, including the ability to understand, predict, and manage the spread of the COVID‐19 virus at the local, regional, and national levels, with ensuing impact on individual and population health. However, due to a number of sociocultural, policy, legal, and technical barriers and “gaps,” implementing this type of data sharing and collaborative analytics is both challenging and extremely costly from a time and resource perspective.

We believe that a number of systematic steps can be taken to address such barriers, based in part on our lessons learned at Washington University and BJC HealthCare. While by no means a comprehensive evaluation of such issues, we envision that many of our experiences are shared at a national level, and further, can serve as the catalyst for a robust community‐wide dialogue on what steps can and should be taken to ensure that our regional and national healthcare systems can truly learn, in a rapid manner, so as to respond to this and future emergent public health crises.

## CONFLICT OF INTEREST

The authors have no relevant conflicts of interest to declare.
